# Treatment with Hydroxychloroquine vs Hydroxychloroquine + Nitazoxanide in COVID-19 patients with risk factors for poor prognosis: A structured summary of a study protocol for a randomised controlled trial

**DOI:** 10.1186/s13063-020-04448-2

**Published:** 2020-06-08

**Authors:** José Meneses Calderón, Hugo Mendieta Zerón, Srivatsan Padmanabhan

**Affiliations:** 1 Internal Medicine, Intensive Care Unit, “Lic. Adolfo López Mateos” General Hospital, Toluca, Mexico; 2 “Mónica Pretelini Sáenz” Maternal-Perinatal Hospital, Toluca, Mexico; 3 Autonomous University of the State of Mexico (UAEMex), Internal Medicine and Medical Sciences (UNAM), Endocrinology, University of Santiago de Compostela, Santiago de Compostela, Spain; 4 Faculty of Medicine, UAEMéx, Toluca, Mexico; 5 Research Department, “Mónica Pretelini Sáenz” Maternal-Perinatal Hospital, Toluca, Mexico; 6 Internal Medicine, St. Joseph Medical Center, Tacoma, WA USA

**Keywords:** COVID-19, Randomised controlled trial, protocol, Dual therapy, hydroxychloroquine, nitazoxanide, SARS-CoV-2, treatment regimen

## Abstract

**Objectives:**

To determine the efficacy of Hydroxychloroquine vs. Hydroxychloroquine + Nitazoxanide in reducing the need for invasive mechanical ventilatory support for patients with COVID-19. Hydroxychloroquine is currently being used in multiple trials with varying doses in an attempt to treat COVID-19. Nitazoxanide has powerful antiviral effects and proven efficacy against a range of viruses including SARS and MERS. Dual therapy by combining appropriate doses of these two medications with diverse activities against COVID-19 is expected to be better than monotherapy with hydroxychloroquine.

**Trial design:**

This is a single centre, randomized, controlled, single blinded, 2 arm (ratio 1:1) parallel group trial.

**Participants:**

86 COVID-19 positive patients that are being treated at the Health Institute of the State of Mexico (ISEM) in Toluca, State of Mexico will be recruited from May 14 to December 31, 2020.

Inclusion criteria:

1. Age older than 18 years.
2. Hospitalised COVID-19 PCR test positive patients.
3. Within the first 72 hours after performing the PCR test.
4. Presence of risk factors for complications (at least one): over 60 years, history of diabetes mellitus, hypertension, and morbid obesity.

Exclusion criteria:

1. Patients with corrected QT interval (QTc) greater than 500ms at hospital admission.
2. Patients who have inherent contraindications to each drug.
3. Patients who are unable to consent.
4. Patients who have previously received chloroquine.
5. Patients already intubated.

Elimination criteria:

1. Patients whose clinical follow-up is lost or who decide not to continue in the study

**Intervention and comparator:**

The two management alternatives will be:

Control - Hydroxychloroquine 200 mg taken orally every 12 hours for 7 days.

Dual therapy - Hydroxychloroquine 400 mg taken orally every 12 hours for two days and then 200 mg taken orally every 12 hours for four days + Nitazoxanide 500 mg orally every 6 hours taken with food, for seven days.

**Main outcomes:**

Primary: Mechanical ventilation requirement assessed at one week.

Percentage of COVID-19 positive patients who require mechanical ventilation . All patients will be monitored till hospital discharge or death.

**Randomisation:**

Patients will be randomly allocated using allocation papers and opaque sealed envelopes to either receive the placebo or the dual therapy intervention treatment in a 1:1 ratio until we have recruited the required number of patients for each group.

**Blinding (masking):**

Trial participants will be blinded.

**Numbers to be randomised (sample size):**

86 participants will be randomized to each group, with 43 in the control group and 43 in the dual therapy group.

**Trial Status:**

Protocol version: 2, recruitment will begin on May 14 until sample size is reached , with the analysis deadline of December 31st 2020.

**Trial registration:**

ClinicalTrials.gov Identifier: NCT04341493.

Date of trial registration: April 10, 2020

**Full protocol:**

The full protocol is attached as an additional file, accessible from the Trials website (Additional file 1). In the interest of expediting dissemination of this material, the familiar formatting has been eliminated; this Letter serves as a summary of the key elements of the full protocol

## Supplementary information

**Additional file 1.** Full study protocol.

## Data Availability

The three researchers will have access to the final trial dataset. There are no contractual agreements that limit such access for investigators and will be available on request.

