# Supplementary material for: Treatment with Hydroxychloroquine vs Hydroxychloroquine + Nitazoxanide in COVID-19 patients with risk factors for poor prognosis: A structured summary of a study protocol for a randomised controlled trial
Source: Trials. 2020 Jun 8;21:504. doi: 10.1186/s13063-020-04448-2 (PMC7276957; doi:10.1186/s13063-020-04448-2)
Supplement: Supplementary file 1 — Additional file 1. Full study protocol. [file 13063_2020_4448_MOESM1_ESM.docx]

**Title**

Treatment with Hydroxychloroquine vs Hydroxychloroquine + Nitazoxanide in COVID-19 patients with risk factors for poor evolution

**Names protocol contributors**

José Meneses Calderón M.D.

Hugo Mendieta Zerón M.D., Ph.D.

Srivatsan Padmanabhan M.D., Ph.D.

**Abstract**

- **Background**: The current pandemic caused by the SARS-CoV2 coronavirus (COVID-19) is life-threatening and is challenging the world's best health systems. None of the novel therapeutics that are being studied worldwide will be accessible immediately and in a broad way. The aim of this project is to repurpose a combination of two widely available medications with known *in vitro* activity and compare the effect of hydroxychloroquine alone versus the combination of nitazoxanide to the hydroxychloroquine against SARS-CoV-2.
- **Methods**: This clinical trial will be performed in Toluca, State of Mexico, from April to December, 2020. Patients who test positive for COVID-19 by qPCR or those who fulfill the clinical criteria will be invited to participate and receive either of two treatments: a) Hydroxychloroquine 200 mg PO every 12 hours for 7 days, b) Hydroxychloroquine 400 mg PO every 12 hours for two days and then 200 mg PO every 12 hours for four days + nitazoxanide 500 mg PO every 6 hours for seven days.
- **Discussion**: Hydroxychloroquine is currently being used in multiple trials with varying doses in an attempt to treat COVID-19. However, it is important to achieve rapid and adequate tissue concentrations for therapeutic effect. Moreover, Nitazoxanide has powerful antiviral effects through its ability to amplify the interferon pathway including an increase in phosphorylated factor 2-alpha, an intracellular protein with antiviral effects. Its efficacy has been tested against 16 strains of influenza A / H1N1, H3N2, H3N2v, H3N8, H5N9, H7N1 and a strain of influenza B. It has also demonstrated efficacy against respiratory syncytial virus, norovirus, dengue, yellow fever, Japanese encephalitis virus, rotavirus, hepatitis B and C, HIV, SARS and MERS. Dual therapy by combining these two medications with diverse activities against COVID-19 is expected to be better than monotherapy with hydroxychloroquine. If efficacy is proven against SARS-CoV-2 this combination therapy will be an excellent therapeutic option for large scale intervention due to the low-cost and wide availability.
- **Trial registration**: ClinicalTrials.gov Identifier: NCT04341493.

**Keywords**

Hydroxychloroquine, nitazoxanide, SARS-CoV-2.

**Administrative information**

| Title {1} | Treatment with hydroxychloroquine vs nitazoxanide + hydroxychloroquine in patients with COVID-19 with risk factors for poor outcomes |
| --- | --- |
| Trial registration {2a and 2b}. | ClinicalTrials.gov Identifier: NCT04341493. |
| Protocol version {3} | Version 2: 22-04-2020. |
| Funding {4} | SPIRIT guidance: Sources and types of financial, material, and other support. |
| Author details {5a} | José Meneses Calderón  “Mónica Pretelini Sáenz” Maternal-Perinatal Hospital  Hugo Mendieta Zerón  “Mónica Pretelini Sáenz” Maternal-Perinatal Hospital  Srivatsan Padmanabhan  St. Joseph Medical Center |
| Name and contact information for the trial sponsor {5b} | Hugo Mendieta Zerón  Felipe Villanueva sur 1209. Col. Rancho Dolores. 50170. Toluca, Mexico. E-mail: drmendietaz@yahoo.com |
| Role of sponsor {5c} | The sponsor is the Chief of the Research Department, “Mónica Pretelini Sáenz” Maternal-Perinatal Hospital, his role is to lead the administrative coordination to get the supplies for the study |

**Introduction**

**Background and rationale {6a}**

Coronaviruses (CoV) are positive-sense single-stranded RNA viruses in the Coronaviridae family (Coronavirinae subfamily) that infect a wide range of hosts to produce diseases ranging from the common cold to serious / fatal diseases [1]. The current pandemic caused by the SARS-CoV2, named coronavirus disease (COVID-19) is life-threatening and is challenging the world's best health systems [2,3]. Based on the large number of infected people who were exposed to the wet animal market in Wuhan City, China, COVID-19 is likely of zoonotic origin [4].

Rapid spread and the ensuing havoc have led physicians to try a variety of treatments without adequate evidence and without clear understanding of the disease [5]. The mainstay of disease control has remained epidemiological, namely, isolation, quarantine measures and other deep cleaning strategies [6]. Current epidemiological data suggests that special attention and efforts should be taken to protect or reduce transmission in susceptible populations, including the elderly or those with comorbidities [7]. Moreover, there is a paucity of information about COVID-19 in pregnant women [8]. The case fatality rate ranges from 1 to 2.2% [3] with significant differences depending on the affected country [9].

**Evolution**

By April 24 of this year, there were 2,729,274 cases worldwide, with 191,127 confirmed cases and 191,614 deaths [10]. In Mexico, 19,924 cases have been confirmed, with 1,859 deaths [11]. Based on current capacity of public hospitals in most states there is reason to believe that the numbers above are likely much higher due to under diagnosis. However, deaths in Mexico show a less pronounced upward curve than in severely affected countries, which gives us a window of opportunity to act before the problem overflows in our hospitals.

By the calculations of the Ministry of Health (Mexico) around 6% of the patients (approximately 10,500) who contract COVID-19 could become serious and in need of hospitalization in intensive care. If only 3,000 beds are counted, then more than 6,500 patients would have no option to receive intensive care [12].

With the current pandemic accelerating, the death toll continues to accumulate [13] while the process to get an effective vaccine is still in the future without a definitive date [14]. Similar issues plague the design and approval of novel therapeutics. Time is of the essence in reducing the human life and economic toll of this pandemic. Repurposing drugs with proven safety record based on clear scientific principles offers an excellent option against this pandemic [15].

**Treatment**

Treating coronavirus infections such as Severe Acute Respiratory Syndrome (SARS), Middle East Respiratory Syndrome (MERS) and COVID-19 have been challenging. A wide range of drugs have been used but none have been shown to have clear evidence of benefit. Following are some examples of therapy used for COVID-19:

1. Direct antiviral therapy – including HIV protease inhibitors have been attempted. Based on data analyzed retrospectively in cases of SARS earlier treatment showed better results [16-19]. In the various series in the literature, this combination of lopinavir / ritonavir (LPV / r) associated with ribavirin can cause serious side effects in a low percentage of cases, consisting of hypersensitivity reactions, Stevens-Johnson syndrome, cardiac, metabolic, hepatic, hematological, and pancreatic complications [20]. Other antiviral agents such as darunavir, favipiravir, galidesivir, nelfinavir and arbidol have also been used [21].

2. Chloroquine and hydroxychloroquine, originally an antimalarial agent with immunosuppressive activity and showing antiviral activity *in vitro*. It interferes with glycosylation of angiotensin-converting enzyme 2 (ACE2) receptors as well as with the cellular internalization of the virus [19, 22]. It can have severe adverse reactions on the cardiac conduction system, arrhythmias, seizures, anaphylaxis reactions, neuromuscular disorders, neuropsychiatric disorders, liver toxicity, and hematological abnormalities [23].

3. Steroids. High doses of hydrocortisone showed reduced expression of pro-inflammatory CXCL8 and CXCL10 chemokines in intestinal cells infected with the SARS coronavirus [24]. However, without an effective antiviral agent, high doses of steroids for prolonged periods has detrimental effects, among others, increases the viral load, spread as well as increasing the risk of nosocomial infections [25, 26].

4. Remdesivir – an adenosine analogue originally developed against Ebola virus [27] but also shown to work against SARS-CoV-2, acts by interfering with replication by causing premature termination of RNA polymerization [28].

5. Convalescent plasma transfusion [29-31].

6. An extensive and vast list that includes small molecules of which there are twelve patents under investigation and 19 antiviral antibodies [32, 33].

The multinational project called “Solidarity” [34] by WHO is currently testing the following four treatments in an adaptive trial:

1. Remdesivir. With no confirmatory results [35] it has been approved by the FDA.

2. Chloroquine / hydroxychloroquine. Were included in Chinese Clinical Practice Guidelines.

3. Ritonavir and lopinavir. HIV protease inhibitor which in preliminary trials have not shown encouraging results against SARS-CoV-2.

4. Ritonavir / lopinavir and interferon-beta. The combination of these have been shown to be effective in animals infected with MERS.

Unfortunately, not all of these options will be available immediately and widely for patients in Mexico or in most Developing countries.

Proposing combination therapy of hydroxychloroquine and nitazoxanide

Nitazoxanide

Nitazoxanide (NTZx) is a derivative of 5-nitrothiazole, synthesized in 1974 by Rosignol – Cavier and patented in 1975 [36]. Its anti-protozoal activity is due to interference with the enzyme pyruvate-ferredoxin oxidoreductase and to a lesser extent with dehydrogenase, which reduces ferredoxin, modifying the anaerobic cellular intermediate metabolism. It has reasonable oral absorption and the concentrations of its active metabolite Tizoxanide is observed by two to three hours in serum. Tizoxanide has a half-life ot 1.5-1.8 h and subsequently is conjugated to gluconoride which is excreted in urine, faeces and bile [37].

NTZx is a drug with antibacterial and anti-protozoal effects, but serendipitously found to have powerful anti-viral effects through multiple mechanisms that amplify host innate immunity/interferon pathway. NTZx amplifies upstream components of interferon pathway such as RIG-1, MDA-5 and also downstream effectors such as increased phosphorylation of PKR (protein kinase activated by double-stranded RNA), MAVS, interferon and multitude of interferon stimulated genes like *Mx2* and *IFITM3* [38]. The anti-viral activity of Nitazoxanide is primarily due to Tizoxanide (desacetylnitazoxanide). The glucuronide conjugate is devoid of antiviral effect. Dosing NTZx appropriately for anti-viral effect is critical for its efficacy in viral infections.

NTZx and its circulating active metabolite Tizoxanide have been shown to inhibit the replication of a wide range of both RNA and DNA viruses. It has antiviral activity against 16 strains of influenza A / H1N1, H3N2, H3N2v, H3N8, H5N9, H7N1 and a strain of influenza B. It has also been shown to have activity against respiratory syncytial virus, norovirus, dengue, yellow fever, Japanese encephalitis virus, rotavirus, hepatitis B and C, human immunodeficiency virus, SARS, and MERS that is consistent with broad spectrum antiviral activity [39].

Studies in 2012 showed that Nitazoxanide was able to suppress MERS virus *in vitro*. NTZx was demonstrated to inhibit viral protein nucleoprotein but also demonstrated beneficial activity of suppressing pro-inflammatory cytokines such as tumor necrosis factor alpha (TFN alpha), interleukin (IL) -2, IL-4, IL-5, IL-6, IL-8 and IL-10, in peripheral blood mononuclear cells, with the administration of NTZx orally and intraperitoneally in animal models [40].

A phase-II study conducted during the influenza pandemic in the United States during the 2010-2011 period was divided into three phases. The first two included patients with severe acute respiratory viral disease and the third with uncomplicated acute influenza of less than 96 hours of evolution. The first phase included 100 children from 1 to 11 years old, 50 managed with NTZx and 50 with placebo. In the second phase there were 86 subjects older than 12 years and adults, 43 in the NTZx group and 43 in the placebo group. The third was integrated with 74 people from 12 to 65 years old, equally divided into two groups. In this phase 63% had positive identification for influenza viruses: A / H1N1 (50%), influenza B (30%) and AH3N2 (20%). Other viruses were rhinovirus (14%) and coronavirus (3%). Study analysis demonstrated a significant reduction in symptom resolution time in the NTZx group [41]. A phase II study was also carried out in six hospitals in Mexico from March-2014 to March-2017 with a methodology similar to the previous study but in this case treatment did not decrease the length of hospital stay in the study population [42]. The lack of efficacy was most likely due to inadequate dosing of NTZx for anti-viral effect [43]

Hydroxychloroquine/chloroquine:

HCQ/CQ acts by reducing glycosylation of ACE2 thereby reducing the binding to the viral spike protein. In addition, chloroquine/HCQ increases the endosomal pH thereby interfering with the fusion of viral membrane to endosomes and trapping viral particles in the endosomes. Time of infection studies also show there may be an independent post-entry mechanism of action that is not fully elucidated(1). Both HCQ and CQ are also known to anti-inflammatory effects including reduced IL6 and associated inflammatory mediators therefore potentially reducing host tissue damage [44]. The combination of HCQ and NTZx is likely to be superior to HCQ alone due to the multitude of diverse mechanisms affected by the combination [45].

**Objectives {7}**

**General**

To determine the efficacy of Hydroxychloroquine vs. Hydroxychloroquine + Nitazoxanide in reducing COVID-19 patients that require invasive mechanical ventilatory support.

**Specific**

Determine the average age of patients who contract COVID-19.

Report the main comorbidities that occur.

Determine the number of cases admitted to an intensive care unit with one or the other treatment.

Monitor and document the evolution of COVID-19

**Hypotheses**

The use of Hydroxychloroquine + Nitazoxanide with in the first 48 hours of diagnosing COVID-19 in high-risk patients will decrease the number of patients who will require invasive mechanical ventilation support by at least 20% in comparison to patients receiving only Hydroxychloroquine.

**Trial design {8}**

This is a parallel group.

**Methods: Participants, interventions and outcomes**

**Study setting {9}**

Place of study: State of Mexico.

Study time: From April 10 to December 31, 2020.

Universe: COVID-19 positive patients treated at the Health Institute of the State of Mexico (ISEM).

**Eligibility criteria {10}**

Inclusion criteria:

1) Age older than 18 years.

2) COVID-19 positive patients.

3) With risk factors for complications (at least one): over 60 years, history of diabetes mellitus, hypertension, and morbid obesity.

Exclusion criteria:

Patients who have inherent contraindications to each drug.

Elimination criteria:

Patients whose clinical follow-up is lost or who decide not to continue in the study.

**Who will take informed consent? {26a}**

The physician caring for each patient will obtain the informed consent.

**Additional consent provisions for collection and use of participant data and biological specimens {26b}**

The participant data will be managed according to The Federal Law on Protection of Personal Data Held by Individuals (LFPDPPP), Mexico. The researcher won`t retain biological samples.

**Interventions**

**Explanation for the choice of comparators {6b}**

Hydroxychloroquine was chosen as referred by the first papers that analysed several drugs to treat COVID-19.

Nitazoxanide was chosen based on the previous *in vitro* activity against SARS, MERS and due to its *in vitro* activity against SARS-CoV-2 in addition to its easy availability and low-cost.

**Intervention description {11a}**

All patients admitted with a diagnosis of a respiratory infection that is confirmed as COVID-19 by PCR or in its absence, a patient who has two major criteria and one minor criterion (Table 1) with high risk factors (age over 60 years, diabetes mellitus or morbid obesity). Patients who meet the inclusion criteria will require signed informed consent followed by randomization by means of allocation papers to receive one or the other treatment until all patients are completed for each group.

The two management alternatives will be:

a) Hydroxychloroquine 200 mg PO every 12 hours for 7 days.

b) Hydroxychloroquine 400 mg PO every 12 hours for two days and then 200 mg PO every 12 hours for four days + NTZx 500 mg PO every 6 hours for seven days.

**Table 1.** Diagnostic Criteria for COVID-19

| **Major criteria** | **Minor criteria** |
| --- | --- |
| Headache  Fever  Cough | Dyspnea  Arthralgia  Myalgia  Odynophagia  Rhinorrhea  Conjunctivitis  Chest pain |

**Criteria for discontinuing or modifying allocated interventions {11b}**

Drug adverse effects, participant request, or if the ISEM runs out of the medicine.

**Strategies to improve adherence to interventions {11c}**

The nurse staff supervises the correct intra-hospital administration of the drugs.

**Relevant concomitant care permitted or prohibited during the trial {11d}**

Management protocol for any underlying disease according to current clinical practice guidelines.

**Provisions for post-trial care {30}**

If any adverse event were to happen, all trial participants will receive medical attention for free in the ISEM

**Outcomes {12}**

Primary: Mechanical ventilation requirement.

Percentage of patients COVID-19 positive that required mechanical ventilation.

**Participant timeline {13}**

|  | **Study period** |  |  |  |  |  |  |  |  |
| --- | --- | --- | --- | --- | --- | --- | --- | --- | --- |
|  | **Enrolment** | **Allocation** | **Post-allocation** |  |  |  |  |  | **Close-out** |
| **TIME POINT**** | ***-t_1_*** | **0** | ***t_1_*** | ***t_2_*** | ***t_3_*** | ***t_4_*** | ***T_5_*** | ***t_6_*** | ***t_6_*** |
| **ENROLMENT:** |  |  |  |  |  |  |  |  |  |
| **Eligibility screen** | X |  |  |  |  |  |  |  |  |
| **Informed consent** | X |  |  |  |  |  |  |  |  |
| ***[List other procedures]*** | X |  |  |  |  |  |  |  |  |
| **Allocation** |  | X |  |  |  |  |  |  |  |
| **INTERVENTIONS:** |  |  |  |  |  |  |  |  |  |
| ***[Intervention A]*** |  |  | X | X | X | X | X | X | X |
| ***[Intervention B]*** |  |  | X | X | X | X | X | X | X |
| **ASSESSMENTS:** |  |  |  |  |  |  |  |  |  |
| ***[List baseline variables]*** | X | X |  |  |  |  |  |  |  |
| ***[List outcome variables]*** |  |  |  | X | X | X | X | X | X |
| ***[List other data variables]*** |  |  | X | X | X | X | X | X | X |

**Sample size {14}**

Sampling method: Probabilistic

Sample size: The following formula is used for proportions:


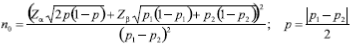


Where Zα = 1,645, Zβ = 1,282. If it is considered that they are improving (avoiding invasive mechanical ventilation support) only 5% of patients with positive COVID-19 risk factors (p2 = 0.05) and seek to increase this improvement by 20%, our p1 is 0.25 and our n will be 43.48 = 43 patients per treatment group.

**Recruitment {15}**

Ten Hospitals of the ISEM have been invited to participate in the study.

**Assignment of interventions: allocation**

**Sequence generation {16a}**

The allocation was computer based.

**Concealment mechanism {16b}**

The allocation sequence will be by central telephone, calling each case to the Research Unit of the “Mónica Pretelini Sáenz” Maternal-Perinatal Hospital (HMPMPS).

**Implementation {16c}**

Hugo Mendieta Zerón will generate the allocation sequence, every COVID Hospital will enroll participants, and the Medical Doctor in direct charge of the patients will assign them to interventions.

**Assignment of interventions: Blinding**

**Who will be blinded {17a}**

Trial participants will be blinded.

**Procedure for unblinding if needed {17b}**

Unblinding is permissible if the patient requests for any adverse event or discomfort due to the treatment.

**Data collection and management**

**Plans for assessment and collection of outcomes {18a}**

The team will be trained in the software ClinCapture and before the collection of outcome, baseline, and other trial data, Dr. José Meneses Calderón and Dr. Hugo Mendieta Zerón will verify the Medical Files.

**Plans to promote participant retention and complete follow-up {18b}**

The feasibility of completing the study is high as all study participants are inpatients and closely monitored until discharge or in an unfortunate event of death.

**Data management {19}**

The medical files of the patient will be kept for five years in every Hospital where the patients are enrolled. To avoid dispersion only one person in Mexico will be in charge of the data entry. Data will be analyzed with SPSS and stored in Excel format.

**Confidentiality {27}**

Personal information will be kept safe in each Statistical Unit of the participant medical units. In no case will the physical files be collected and only the researchers will have access to the information concentrated in electronic databases without mentioning the names of the patients.

**Plans for collection, laboratory evaluation and storage of biological specimens for genetic or molecular analysis in this trial/future use {33}**

Samples will be collected as requested for the usual and necessary information depending on the individual circumstances of the patients. There won`t be storage of biological specimens for genetic or molecular analysis besides that required for the SARS-CoV-2 identification and cure verification.

**Statistical methods**

**Statistical methods for primary and secondary outcomes {20a}**

A descriptive analysis will be performed for all the quantitative variables. To evaluate the differences in the means between the groups, the student's T test will be used for the variables with normal distribution, and the Mann – Whitney U test for the variables with non-normal distribution (non-parametric tests).

Regression studies will be carried out to determine the most important variables that influence the prognosis taking into account both treatment schemes. Due to the nature of the study, estimates will be made of the number necessary to treat (NNT) to avoid reaching a case of ventilatory mechanical support.

A confidence interval of 95% (I.C. at 95%) will be estimated, so the values of p <0.05 will be considered statistically significant. All analyzes will be performed using SPSS version 22 software.

**Interim analyses {21b}**

Dr. Hugo Mendieta Zerón will perform interim analyses and the full team of researchers will have access to this information to make the final decision to terminate the trial.

**Methods for additional analyses (e.g. subgroup analyses) {20b}**

In case of changes in the drug suppliers or difficulties to get them, it will be considered the option to perform additional analyses.

**Methods in analysis to handle protocol non-adherence and any statistical methods to handle missing data {20c}**

As the population will be hospitalized it is expected to have low non-adherence and in case of such a thing, the elimination criteria will be applied. If specific laboratories are found to be absent, they we’ll be treated in SPSS as missing values.

**Plans to give access to the full protocol, participant level-data and statistical code {31c}**

The protocol is expected to have full access to anyone interested. The full data of the patients will be restricted and only available for the researchers involved in this project.

**Oversight and monitoring**

**Composition of the coordinating centre and trial steering committee {5d}**

The coordinating center is composed by two researchers who get the calls from other hospitals of the ISEM to adhere candidate patients. The data management team will be integrated by Hugo Mendieta Zerón from the HMPMPS, Toluca, Mexico and by Srivatsan Padmanabhan from the St Joseph Medical Centre, Tacoma, USA.

**Composition of the data monitoring committee, its role and reporting structure {21a}**

In the HMPMPS, the Ethics in Research Committee (CEI) and the Research Committee (CI) function as Data Monitoring Committees, supervising the partial and final advances in the Research Protocols.

**Adverse event reporting and harms {22}**

The HMPMPS has its own Committee of Adverse Events.

**Frequency and plans for auditing trial conduct {23}**

The HMPMPS belongs to the ISEM and this authority can plan an audit if necessary.

**Plans for communicating important protocol amendments to relevant parties (e.g. trial participants, ethical committees) {25}**

There is a possibility to communicate protocol amendments if, by commercial issues (importation procedures) there is a delay in the administration of any drug used in the trial. If a clear benefit is observed with the use of one of the arms, then all the patients will receive the best treatment.

**Dissemination plans {31a}**

The main objective of the research team is to help physicians identify other treatment options. Trial participants will be informed every day about their progress. We will be using social media, videoconferences to share details about our clinical trial. Transmission will be free for both professionals and the public. The final academic target is to publish the results in an international medical journal.

**Discussion**

Laboratorial and clinical data are critical to guarantee the reliability of the results.

**Trial status**

This is the protocol version number 2 (April 2020), with the corrections suggested by the CEI and the CI. The recruitment will begin on May 7 and will finish in September 2020.

**Abbreviations**

ACE2: angiotensin-converting enzyme 2.

CoV: Coronaviruses.

COVID-19: coronavirus disease.

HCQ: Hydroxychloroquine.

HMPMPS: “Mónica Pretelini Sáenz” Maternal-Perinatal Hospital.

IL: interleukin.

ISEM: Health Institute of the State of Mexico.

LFPDPPP: Federal Law on Protection of Personal Data Held by Individuals.

MERS: Middle East respiratory syndrome.

NTZx: Nitazoxanide.

SARS: Severe acute respiratory syndrome.

TFN alpha: tumor necrosis factor alpha.

**Declarations**

**Acknowledgements**

Pending the evaluation of the participation of more professionals.

**Authors’ contributions {31b}**

JMC: Chief Investigator; he conceived the study, conceptualization, literature review.

HMZ: writing of the protocol and logistics.

SP: contributed to study design and to development of the proposal.

All authors read and approved the final manuscript.

**Funding {4}**

This study has no a specific funding as the medical attention is part of the routine work.

**Availability of data and materials {29}**

The three researchers will have access to the final trial dataset. There is no any contractual agreements that limit such access for investigators.

**Ethics approval and consent to participate {24}**

This project has been authorized (code 2020-03-681) by the Research Ethics Committee of the Maternal-Perinatal Hospital "Mónica Pretelini Sáenz" (HMPMPS), with current registration with the National Bioethics Commission (CONBIOETICA) as well as by the Research Committee of the same Hospital with current registration in the Federal Commission for the Protection against Health Risks (COFEPRIS). It has also been approved by the Research Ethics Committee of the General Hospital of Mexico "Dr. Eduardo Liceaga".

The research will be carried out under the ethical considerations recognized by the Declaration of Helsinki (Fortaleza, Brazil, 2013), and according to the level of intervention, it is considered a study of greater than minimum risk. In any case, an informed consent format will be applied. Written, informed consent to participate will be obtained from all participants.

**Consent for publication {32}**

A model consent form should be available upon request.

**Competing interests {28}**

The authors declare that they have no competing interests.

**Authors’ information (optional)**

JMC: Internal Medicine, Intensivist. Chief of the Intensive Care Unit of the “Lic. Adolfo López Mateos” General Hospital in Toluca, Mexico for 23 years. Former Director of the “Mónica Pretelini Sáenz” Maternal-Perinatal Hospital.

HMZ: Medical Doctor (Autonomous University of the State of Mexico (UAEMex)), Internal Medicine and Master in Medical Sciences (UNAM), PhD in Endocrinology (University of Santiago de Compostela, Spain). He is a Full-Time Professor at the Faculty of Medicine, UAEMéx and Chief of the Research Department at the “Mónica Pretelini Sáenz” Maternal-Perinatal Hospital.

SP: Hospitalist, Internal Medicine, St. Joseph Medical Center, Tacoma, WA, USA.

**References**

1. Dhama K, Sharun K, Tiwari R, Dadar M, Malik YS, Singh KP, et al. COVID-19, an emerging coronavirus infection: advances and prospects in designing and developing vaccines, immunotherapeutics, and therapeutics. Hum Vaccines Immunother. 2020;1-7.
2. Kinross P, Suetens C, Gomes Dias J, Alexakis L, Wijermans A, Colzani E, et al. Rapidly increasing cumulative incidence of coronavirus disease (COVID-19) in the European Union/European Economic Area and the United Kingdom. 2020. Euro Surveill Bull Eur Sur Mal Transm Eur Commun Dis Bull.
3. Bassetti M, Vena A, Giacobbe DR. The novel Chinese coronavirus (2019‐nCoV) infections: Challenges for fighting the storm. Eur J Clin Invest. 2020;50(3).
4. Rothan HA, Byrareddy SN. The epidemiology and pathogenesis of coronavirus disease (COVID-19) outbreak. J Autoimmun. 2020;102433.
5. Pang J, Wang MX, Ang IYH, Tan SHX, Lewis RF, Chen JI-P, et al. Potential Rapid Diagnostics, Vaccine and Therapeutics for 2019 Novel Coronavirus (2019-nCoV): A Systematic Review. J Clin Med. 2020;9(3).
6. Ng Y, Li Z, Chua YX, Chaw WL, Zhao Z, Er B, et al. Evaluation of the Effectiveness of Surveillance and Containment Measures for the First 100 Patients with COVID-19 in Singapore - January 2-February 29, 2020. MMWR Morb Mortal Wkly Rep. 2020;69(11):307-11.
7. Applegate WB, Ouslander JG. COVID-19 Presents High Risk to Older Persons. J Am Geriatr Soc. 2020;
8. Liang H, Acharya G. Novel corona virus disease (COVID-19) in pregnancy: What clinical recommendations to follow? Acta Obstet Gynecol Scand. 2020;99(4):439-42.
9. Sevillano Pires L, Andrino B, Llaneras K, Grasso D. El mapa del coronavirus: así crecen los casos día a día y país por país. Available at: <https://elpais.com/sociedad/2020/03/16/actualidad/1584360628_538486.html>
10. COVID-19 Dashboard by the Center for Systems Science and Engineering (CSSE) at Johns Hopkins University. Available at: <https://coronavirus.jhu.edu/map.html>
11. [Daily Report of COVID]. Available at: <https://coronavirus.gob.mx/>
12. Miranda P. Sector Salud, con 4,291 camas y 2.053 ventiladores para combatir coronavirus. Available at: <https://www.eluniversal.com.mx/nacion/sector-salud-con-4291-camas-y-2053-ventiladores-para-combatir-coronavirus>
13. Putra M, Kesavan MM, Brackney K, Hackney DN, Roosa MKM. Forecasting the Impact of Coronavirus Disease During Delivery Hospitalization: An Aid for Resources Utilization. Am J Obstet Gynecol MFM. 2020:100127. doi: 10.1016/j.ajogmf.2020.100127.
14. Thanh Le T, Andreadakis Z, Kumar A, Gómez Román R, Tollefsen S, Saville M, et al. The COVID-19 vaccine development landscape Nat Rev Drug Discov. 2020 Apr 9. doi: 10.1038/d41573-020-00073-5. [Epub ahead of print]
15. Gordon DE, Jang GM, Bouhaddou M, Xu J, Obernier K, White KM, et al. A SARS-CoV-2 protein interaction map reveals targets for drug repurposing. Nature. 2020 Apr 30. doi: 10.1038/s41586-020-2286-9. [Epub ahead of print].
16. Mo Y, Fisher D. A review of treatment modalities for Middle Respiratory Syndrome. J Antimicrob Chemother 2016;71:3340-3350.
17. Al-Tawfiq JA, Al-Homoud AH, Memish ZA. Remdesivir as a posible therapeutic option for the COVID-19. Travel Med and Infect Dis. 2020 Mar 5:101615. doi: 10.1016/j.tmaid.2020.101615. [Epub ahead of print]
18. Chan KS, Lai ST, Chu CM, Tsui E, Tam CY, Wong MM, et al. Treatment of severe acute respiratory sindrome with lopinavir/ritonavir: A multicentre retrospective matched cohort study. Hong Kong Med J 2003;9:399-406.
19. Chu CM, Cheng VC, Hung IF, Wong MM, Chan KH, Chan KS, et al. Role of lopinavir/ritonavir in the treatment of SARS: initial virological and clinical findings. Thorax 2004;59:252-256.
20. Li H, Wang YM, Xu JY, Cao B. Potential antiviral therapeutics for 2019 novel coronavirus. Zhonghua Jie He He Hu Xi Za 2020;43(3):170-172.
21. Yao TT, Qian JD, Zhu WY, Wang Y, Wang GQ. A systematic review of lopinavir therapy for SARS coronavirus and MERS coronavirus – A possible reference for coronavirus disease-19 treatment option. J Med Virol. 2020 Feb 27. doi: 10.1002/jmv.25729. [Epub ahead of print].
22. Liu C, Zhou Q, Li Y, Garner LV, Watkins SP, Carter LJ, et al. Research and Development on Therapeutic Agents and Vaccines for COVID-19 and Related Human Coronavirus Diseases. ACS Cent Sci. 2020;6(3):315-331.
23. Al-Bari MAA. Targetting endosomal acidification by chloroquine analogs as a promising strategy for the treatment of emerging viral diseases. Pharmacol Res Perspect. 2017 Jan 23;5(1):e00293.
24. Wang M, Cao R, Zhang L, Yang X, Liu J, Xu M, et al. Remdesivir and chloroquine effectively inhibit the recently emerged novel coronavirus (2019-nCoV) in vitro. Cell Res 2020;30:269-273.
25. Ritesh Maharaj. King´s Critical Care – Evidence Summary Clinical Management of COVID-19. King´s College Hospital. 9th March 2020. Available at: <file:///F:/NTXZ/Kings%20College%20Critical%20care>% 20COVID19%20Evidence%20Summary.pdf
26. Cinatl J Jr, Michaelis M, Morgenstern B, Doerr HW. High-dose hydrocortisone reduces expression of the pro-inflamatory chemokines CXCL8 y CXCL10 in SARS coronavirus-infected intestinal cells. Int J Mol Med 2005;15:323-327.
27. Tchesnokov EP, Feng JY, Porter DP, Götte M. Mechanism of Inhibition of Ebola Virus RNA-Dependent RNA Polymerase by Remdesivir. Viruses. 2019 Apr 4;11(4). pii: E326. doi: 10.3390/v11040326.
28. Ferner RE, Aronson JK. Remdesivir in covid-19. BMJ. 2020;369:m1610. doi: 10.1136/bmj.m1610.
29. Lee N, Allen Chan KC, Hui DS, Ng EK, Wu A, Chiu RW, et al. Effects of early corticosteroid treatment on plasma SARS-associated Coronavirus RNA concentrations in adult patients. J Clin Virol 2004;31(4):304-309.
30. Xiao JZ, Ma L, Gao J, Yang ZJ, Xing XY, Zhao HC, et al. Glucocorticoid-induced diabetes in severe acute respiratory syndrome: the impact of high dosage and duration of methylprednisolone therapy. Zhonghua Nei Ke Za Zhi 2004;43(3):179-182.
31. Wong VW, Dai D, Wu AK, Sung JJ. Treatment of severe acute respiratory syndrome with convalescent plasma. Hong Kong Med J 2003;9:199-201.
32. Yeh KM, Chiueh TS, Siu LK, Lin JC, Chan PK, Peng MY, et al. Experience of using convalescent plasma for severe acute respiratory syndrome among healthcare workers in a Taiwan hospital. J Antimicrob Chemother 2005;56:919-922.
33. Cheng Y, Wong R, Soo YO, Wong WS, Lee CK, Ng MH, et al. Use of convalescent plasma therapy in SARS patients in Hong Kong. Eur J Clin Microbiol Infect Dis 2005;24:44-46.
34. “Solidarity” clinical trial for COVID-19 treatments. Available at: https://www.who.int/emergencies/diseases/novel-coronavirus-2019/global-research-on-novel-coronavirus-2019-ncov/solidarity-clinical-trial-for-covid-19-treatments
35. Mahase E. Covid-19: Remdesivir is helpful but not a wonder drug, say researchers BMJ. 2020 May 1;369:m1798. doi: 10.1136/bmj.m1798.
36. Rossignol JF, Cavier R. 2-benzamido 5-nitrothiazoles. Chemical Abstract. 1975; 83: 28216n and New Derivatives of 2-Benzamido 5-Nitrothiazoles. United States Patent No 3,950,351, April 13, 1976.
37. Broekhuysen J, Stockis A, Lins RL, De Graeve J, Rossignol JF. Nitazoxanide: pharmacokinetics and metabolism in man Int J Clin Pharmacol Ther. 2000;38(8):387-94.
38. Jasenosky LD, Cadena C, Mire CE, Borisevich V, Haridas V, Ranjbar S, et al. The FDA-Approved Oral Drug Nitazoxanide Amplifies Host Antiviral Responses and Inhibits Ebola Virus. iScience. 2019 Sep 27;19:1279-1290.
39. Cheng VC, Lau SK, Woo PC, Yuen KY. Severe Acute Respiratory Syndrome Coronavirus as an Agent of Emerging and reemerging Infection. Clin Microbiol Reviews 2007;20(4):660-694.
40. Rossignol JF. Nitazoxanide: a first-in-class broad-spectrum antiviral agent. Antiviral Res 2014; 110:94–103) (Rossignol JF. Nitazoxanide, a new drug candidate for the treatment of Middle East respiratory syndrome coronavirus. J Infect Public Health. 2016; 9(3):227–30.
41. A Randomized Double-Blind Phase 2 Study Comparing the Efficacy, Safety, and Tolerability of Nitazoxanide Versus Placebo in Addition to Standard Care for the Treatment of Hospitalized Subjects with Severe Acute Respiratory Illness Sponsored by: Office of Clinical Research Policy and Regulatory Operations (OCRPRO) Division of Clinical Research (DCR) National Institute of Allergy and Infectious Diseases (NIAID). Bethesda. Version 5. Sept 12-2016. Available at: https://clinicaltrials.gov/ProvidedDocs/57/NCT02057757/Prot_001.pdf.
42. Gamiño-Arroyo AE, Guerrero ML, McCarthy S, Ramírez-Venegas A, Llamosas-Gallardo B, Galindo-Fraga A, et al; Mexico Emerging Infectious Diseases Clinical Research Network (LaRed). Efficacy and Safety of Nitazoxanide in Addition to Standard of Care for the Treatment of Severe Acute Respiratory Illness. Clin Infect Dis 2019:69(11):1903-1911.
43. Padmanabhan S, Padmanabhan K. Nitazoxanide - a potential ally in the treatment of COVID-19. Preprint. (2020). published online April 24. DOI: 10.13140/RG.2.2.22854.83527
44. Sinha N, Balayla G. Hydroxychloroquine and covid-19. Postgrad Med J. 2020 Apr 15. pii: postgradmedj-2020-137785. doi: 10.1136/postgradmedj-2020-137785. [Epub ahead of print]
45. Padmanabhan S. Potential dual therapeutic approach against SARS-CoV-2/COVID-19 with Nitazoxanide and Hydroxychloroquine. Preprint. 2020; published online March 15. DOI: 10.13140/RG.2.2.28124.74882
